# Trajectories of body mass index in adulthood and risk of subtypes of postmenopausal breast cancer

**DOI:** 10.1186/s13058-023-01729-x

**Published:** 2023-10-28

**Authors:** Marit Busund, Giske Ursin, Eiliv Lund, Tom Wilsgaard, Charlotta Rylander

**Affiliations:** 1 Department of Community Medicine, Faculty of Health Sciences, UiT The Arctic University of Norway, 9037 Tromsø, Norway; 2 Cancer Registry of Norway, Oslo, Norway

**Keywords:** Breast cancer subtypes, Body fatness, Body mass index, Trajectory, Women

## Abstract

**Background:**

Body fatness is a dynamic exposure throughout life. To provide more insight into the association between body mass index (BMI) and postmenopausal breast cancer, we aimed to examine the age at onset, duration, intensity, and trajectories of body fatness in adulthood in relation to risk of breast cancer subtypes.

**Methods:**

Based on self-reported anthropometry in the prospective Norwegian Women and Cancer Study, we calculated the age at onset, duration, and intensity of overweight and obesity using linear mixed-effects models. BMI trajectories in adulthood were modeled using group-based trajectory modeling. We used Cox proportional hazards models to calculate hazard ratios (HRs) with 95% confidence intervals (CIs) for the associations between BMI exposures and breast cancer subtypes in 148,866 postmenopausal women.

**Results:**

A total of 7223 incident invasive postmenopausal breast cancer cases occurred during follow-up. Increased overweight duration and age at the onset of overweight or obesity were associated with luminal A-like breast cancer. Significant heterogeneity was observed in the association between age at overweight and overweight duration and the intrinsic-like subtypes (*p*_heterogeneity_ 0.03). Compared with women who remained at normal weight throughout adulthood, women with a descending BMI trajectory had a reduced risk of luminal A-like breast cancer (HR 0.54, 95% CI 0.33–0.90), whereas women with ascending BMI trajectories were at increased risk (HR 1.09; 95% CI 1.01–1.17 for “Normal-overweight”; HR 1.20; 95% CI 1.07–1.33 for “Normal-obesity”). Overweight duration and weighted cumulative years of overweight and obesity were inversely associated with luminal B-like breast cancer.

**Conclusions:**

In this exploratory analysis, decreasing body fatness from obesity in adulthood was inversely associated with overall, hormone receptor-positive and luminal A-like breast cancer in postmenopausal women. This study highlights the potential health benefits of reducing weight in adulthood and the health risks associated with increasing weight throughout adult life. Moreover, our data provide evidence of intrinsic-like tumor heterogeneity with regard to age at onset and duration of overweight.

**Supplementary Information:**

The online version contains supplementary material available at 10.1186/s13058-023-01729-x.

## Background

Breast cancer is a heterogeneous disease consisting of at least five distinct molecular subtypes with different etiological pathways and prognosis [1–6]. Owing to the large degree of overlap between these intrinsic subtypes and immunohistochemical subtypes defined by the estrogen receptor (ER), progesterone receptor (PR), and human epidermal growth factor receptor 2 (HER2), the St. Gallen International Expert Consensus panel created an intrinsic-like surrogate definition that has been broadly used in epidemiological research [7]. This classification includes four subtypes: luminal A-like and luminal B-like subtypes, which are predominantly hormone receptor-positive (i.e., ER-positive [ER+] and/or PR-positive [PR+]), and hormone receptor-negative HER2-enriched and basal-like (herein referred to as triple-negative [TNBC]) subtypes.

The risk factors for breast cancer include amongst others exposure to endogenous and exogenous female sex hormones. Hormonal risk factors are associated with hormone receptor-positive and luminal A-like subtypes [8–10]. Less is known about the risk factors for the remaining intrinsic-like subtypes. Adult body fatness, hereafter encompassing overweight and/or obesity, reflects endogenous estrogen exposure through increased aromatization of estrogen precursors in adipose tissue [11–13]. High adult body mass index (BMI) and weight gain are primarily associated with hormone receptor-positive subtypes in postmenopausal women [12, 14–22] and predominantly in never-users of menopausal hormone therapy (MHT) [15–18, 20, 21]. Correspondingly, postmenopausal weight loss reduces the risk of breast cancer among women not using MHT [23–25].

Body fatness is not a static measure but varies over a lifetime, and every woman follows her unique exposure trajectory throughout life. These dynamic aspects are likely relevant for disease development, and such a trajectory approach may provide more insight into the relationship between lifetime exposure intensity, duration and onset, and cancer risk than studying only one or a few measures of exposure. Recent studies have suggested a clear dose–response association between the intensity and duration of body fatness and risk of postmenopausal breast cancer [26, 27]. However, only four previous studies have assessed body fatness trajectories in relation to breast cancer risk [28–31], and only one of them provided estimates based on hormone receptor status [29]. To our knowledge, no previous study has assessed BMI trajectories and the risk of intrinsic-like breast cancer subtypes.

Thus, we aimed to explore whether the intensity, timing, duration, and trajectories of body fatness throughout adult life were associated with breast cancer in postmenopausal women, and whether associations varied according to subtypes.

## Methods

### Study population

The Norwegian Women and Cancer (NOWAC) study is a nationally representative prospective cohort study initiated in 1991 to investigate cancer etiology among women in Norway. Women aged 30–70 years were randomly sampled from the National Population Register and invited to participate in the study. A total of 172,472 women were enrolled between 1991 and 2007 and completed up to three follow-up questionnaires (1998–2017) distributed 5–10 years apart. The unique national identification number assigned to every resident in Norway allows for complete follow-up through linkages to national registries [32]. The NOWAC study is considered to have high external validity, as the distribution of exposures is independent of the response rate, and the cumulative incidence of cancers is similar to national figures from the Cancer Registry of Norway [33]. The details of the NOWAC study have been described previously [34].

In this study, 164,316 women with at least two self-reported height and weight measurements were eligible for inclusion (*n* = 8156 excluded). As physical activity and tobacco smoking affect weight change and hence fluctuations in BMI, we excluded 6697 women without information on these covariates in any of the questionnaires. Excluded women with less than two BMI measurements or missing information on physical activity and tobacco smoking on all time points were slightly older, had higher BMI and lower education than included women (Additional file 1: Table 1). We further excluded women with prevalent cancer (other than non-melanoma skin cancer) at start of follow-up and women diagnosed with cancer within 1 year of the first self-reported weight measurement (*n* = 8150), women who had died or emigrated before start of follow-up (*n* = 457), and women who reported implausible values for age at menarche (< 8 or > 20; *n* = 30), age at menopause (< 25 or > 60; *n* = 111), or age at first birth (< 12 or > 50; *n* = 5). For the complete-case analyses, women with missing covariates were also excluded (*n* = 6095). Thus, the final analytical study sample consisted of 148,866 women, of which 142,771 were included in the complete-case analyses.

### Exposure and covariates assessment

Self-reported weights at age 18 years and at the first, second, and third questionnaires (wave 1–3) and height at wave 1 were used to calculate BMI at up to four time points. As weight loss can follow a cancer diagnosis, weight measurements were not considered valid in women who were diagnosed with cancer up to 1 year before returning the questionnaire. BMI was calculated as weight in kilograms divided by the square of height in meters. Body fatness was defined according to the World Health Organization’s definition [35].

Relevant covariates were extracted from the wave 1 questionnaires. We used a directed acyclic graph to visualize the assumed causal relationships among the exposure, outcome, and covariates, thereby identifying confounding factors to be included in the multivariable regression analysis (Additional file 1: Fig. 1). Identified confounders included age (used as time metric), age at menarche (continuous), parity (0, 1, 2, ≥ 3), age at first birth (< 25, 25–29, ≥ 30 years), history of breast cancer in mother (yes, no), physical activity (low, moderate, high), smoking status (current, former, never), and MHT use (current, former, never). Number of missing values for the covariates are presented in footnotes in Table 1.

**Table 1:** Characteristics of the study sample at wave 1 according to trajectory group

Total study sample	T1 «Normal-stable»^a^	T2 «Normal-overweight»^a^	T3 «Normal-obesity»^a^	T4 «Overweight- obesity»^a^	T5 «Obesity-decrease»^a^
*N* = 148,866	Mean ± SD or *n* (%)	Mean ± SD or *n* (%)	Mean ± SD or *n* (%)	Mean ± SD or *n* (%)	Mean ± SD or *n* (%)
**Wave 1 characteristics**					
Number of participants	65,507 (44.0)	60,440 (40.6)	18,117 (12.2)	3609 (2.4)	1193 (0.8)
Age at enrollment (yrs)	49.1 ± 0.03	49.2 ± 0.03	48.5 ± 0.06	48.2 ± 0.14	53.9 ± 0.26
Age at menarche (yrs)	13.5 ± 0.01	13.2 ± 0.01	12.9 ± 0.01	12.6 ± 0.02	13.1 ± 0.04
Parity	2.1 ± 0.00	2.3 ± 0.01	2.3 ± 0.01	2.2 ± 0.02	2.3 ± 0.04
Age at first birth (yrs)	24.4 ± 0.02	23.8 ± 0.02	23.6 ± 0.04	23.5 ± 0.08	23.9 ± 0.14
Breast cancer in mother	3572 (5.5)	3178 (5.3)	926 (5.1)	182 (5.4)	67 (5.6)
*OC use*					
Current	1261 (2.0)	1142 (1.9)	318 (1.8)	81 (2.3)	4 (0.3)
Former	36,961 (57.7)	32,369 (54.9)	9445 (53.4)	1705 (48.7)	459 (39.7)
Never	25,894 (40.4)	25,508 (43.2)	7922 (44.8)	1713 (49.0)	692 (59.9)
*MHT use*					
Current	8949 (13.9)	7349 (12.4)	1722 (9.7)	258 (7.3)	206 (17.5)
Former	7415 (11.5)	7166 (12.1)	2112 (11.9)	390 (11.0)	141 (12.0)
Never	47,946 (74.6)	44,965 (75.6)	13,964 (78.5)	2896 (81.7)	832 (70.6)
*Smoking status*					
Current	20,984 (32.2)	17,985 (29.9)	5101 (28.3)	993 (27.6)	518 (43.6)
Former	21,243 (32.6)	21,780 (36.2)	6585 (36.5)	1316 (36.6)	322 (27.1)
Never	23,033 (35.3)	20,441 (34.0)	6368 (35.3)	1286 (35.8)	349 (29.4)
*Physical activity*					
High	13,826 (21.3)	10,031 (16.7)	2381 (13.3)	358 (10.0)	243 (20.8)
Moderate	38,527 (59.3)	34,790 (58.0)	9269 (51.6)	1591 (44.6)	633 (54.1)
Low	12,617 (19.4)	15,141 (25.3)	6315 (35.2)	1621 (45.4)	295 (25.2)
*Education (yrs)*					
≤ 9	11,591 (18.5)	13,333 (23.1)	4598 (26.5)	978 (28.3)	423 (39.1)
10–12	20,531 (32.8)	20,422 (35.4)	6288 (36.3)	1267 (36.7)	381 (35.3)
13–16	18,950 (30.3)	15,970 (27.7)	4481 (25.9)	831 (24.1)	199 (18.4)
≥ 17	11,472 (18.3)	7948 (13.8)	1959 (11.3)	379 (11.0)	78 (7.2)
**BMI variables**					
BMI at age 18 (kg/m^2^)	19.7 ± 0.01	21.1 ± 0.01	22.3 ± 0.02	24.9 ± 0.06	34.1 ± 0.10
BMI at wave 1 (kg/m^2^)	21.4 ± 0.01	25.0 ± 0.01	29.6 ± 0.02	36.0 ± 0.07	25.4 ± 0.11
BMI at wave 2 (kg/m^2^)	21.8 ± 0.01	25.7 ± 0.01	30.4 ± 0.02	36.5 ± 0.08	25.6 ± 0.13
BMI at wave 3 (kg/m^2^)	22.1 ± 0.01	26.1 ± 0.02	30.9 ± 0.03	37.1 ± 0.11	26.2 ± 0.23
**Predicted BMI variables**^*b*^					
Age at overweight onset (yrs)	N/A	46.5 ± 0.03	32.5 ± 0.04	21.9 ± 0.07	18.0 ± 0.01
Age at obesity onset (yrs)	N/A	N/A	50.5 ± 0.06	35.9 ± 0.10	19.5 ± 0.24
Overweight duration (yrs)	N/A	7.8 ± 0.03	23.1 ± 0.06	32.9 ± 0.13	35.4 ± 0.35
Obesity duration (yrs)	N/A	N/A	4.6 ± 0.04	18.9 ± 0.14	8.4 ± 0.37
Overweight intensity (OWY)	N/A	10.1 ± 0.01	72.5 ± 0.34	206.9 ± 1.61	123.6 ± 2.65
Obesity intensity (OBY)	N/A	N/A	6.7 ± 0.10	76.4 ± 1.04	14.2 ± 0.92

*BMI* body mass index, *MHT* menopausal hormone therapy, *N/A* not applicable, *OBY* weighted cumulative overweight years, *OC* oral contraceptives, weighted cumulative obesity years, *SD* standard deviation

Number of missing values: 1972 (1.30%) for age at menarche, 9 (0.01%) for parity and age at first birth, 3410 (2.25%) for OC use, 2679 (1.77%) for MHT use, 573 (0.38%) for smoking status, 1232 (0.81%) for physical activity, 6845 (4.52%) for education

^a^Normal weight: 18.5–24.9 kg/m^2^; overweight: 25–29.9 kg/m^2^; obesity: ≥ 30 kg/m^2^

^b^Derived from linear mixed-effects modeling and based on predicted BMI values

### Outcome ascertainment

Incident invasive breast cancer cases were identified through linkage to the Cancer Registry of Norway based on the personal identification number assigned to all Norwegians at birth or immigration, and were classified according to the International Classification of Diseases 10th Revision (ICD-10, C50). Information on death and emigration was obtained through linkage to the Cause of Death Registry and the Central Population Register, respectively.

#### Tumor receptor status

The Cancer Registry of Norway provides information on ER, PR, and HER2 status, assessed using immunohistochemistry (IHC) techniques by pathological departments nationwide. ER negativity was defined as < 10% reactivity before January 2012. From February 2012 onward, the threshold for ER-negative tumors was changed to < 1% reactivity due to changes in the national treatment guidelines. These official thresholds were used in this study. PR negativity was defined as < 10% reactivity. The HER2 expression status was determined using IHC and/or in situ hybridization (ISH). Tumors with no or weak immunostaining were defined as HER2−, while moderate or strong immunostaining was considered HER2 + . ISH was generally used to confirm moderate staining. Breast cancer subtypes were defined by IHC surrogates for molecular subtypes according to the St. Gallen 2013 criteria without using the proliferation marker Ki67 in the subtype definition: luminal A-like (ER + PR + HER2−), luminal B-like (ER + PR− HER2− or ER + PR− HER2 + or ER + PR + HER2 +), HER2-enriched (ER− PR− HER2 +), and TNBC (ER− PR− HER2−) [7].

### Menopausal status

Menopausal status was determined based on reported menstrual history. A woman was considered postmenopausal if her menstrual period had ceased naturally or by bilateral oophorectomy. Age at menopause was defined as the age when menstruation stopped. Women with unknown menopausal status or irregular menstrual cycles were considered postmenopausal at 53 years or older. This cutoff has been used previously in the NOWAC study and is based on the Million Women’s Study convention [36, 37], as 92% and 96% of the study sample aged ≥ 53 years who had not had a hysterectomy or used MHT were postmenopausal, respectively. For women who were current smokers, the age of 53 years was substituted with 51 years, as smoking can reduce the age of menopause onset by approximately 2 years [38]. Menopausal status can be masked by a simple hysterectomy or by using MHT before natural menopause; therefore, women in this category were also considered postmenopausal at age 53 years or older. Women were included in the analysis if they were postmenopausal at the start of follow-up or from the age they reached menopause during the follow-up period.

### Statistical analyses

#### BMI variable constructions

To construct variables for age at onset, duration, and intensity of overweight/obesity, we modeled individual BMI trajectories for each study participant as a function of age, physical activity (time-varying), and tobacco smoking (time-varying) [27, 39] using a linear mixed-effects model with a cubic effect of age and with random intercepts and slopes. As the number of samples was considerably larger than the number of measurement occasions, no assumptions were made regarding the covariance pattern of the random effect; therefore, we fitted an unstructured covariance matrix [40]. For each participant, we interpolated the BMI for each year starting from age 18 years until the last valid BMI measurement. From the predicted values, we calculated the years spent with a BMI ≥ 25 or ≥ 30, hereafter referred to as overweight and obesity durations, respectively. The duration variables did not necessarily reflect consecutive years of overweight/obesity. Furthermore, we calculated the age at first onset of overweight or obesity from age 18 years. Finally, the weighted cumulative overweight years (OWY) and obesity years (OBY) were computed as measures of intensity by multiplying the duration of overweight/obesity in years by the difference (in BMI units) above the normal BMI (≥ 25 kg/m^2^) for overweight and above overweight (≥ 30 kg/m^2^) for obesity for each increment of age. Overweight and obesity duration were assessed per 10-year increments and intensity per 100 units, as previously described [26, 27].

Fluctuations in BMI from age 18 years to the age at the last valid BMI measurement were characterized using Nagin’s approach to group-based trajectory modeling (GBTM) [41, 42]. GBTM is a semiparametric finite mixture model that allows the definition of relatively homogeneous clusters of BMI evolution over age. Trajectories were constructed using a censored normal model in the Traj package in STATA, and the optimal number of groups and shapes of trajectories were evaluated by the Bayesian information criterion using a two-stage approach [43]. First, the number of groups was determined using a quadratic form for all the trajectory groups. Second, the shape of each trajectory was determined. Using this method, the BMI development among the participants was best described by five-group trajectories based on a cubic function of age and adjusted for time-varying physical activity and tobacco smoking covariates. Finally, the average posterior probability and odds of correct classification were calculated, yielding satisfactory results that demonstrated high assignment accuracy based on Nagin’s criteria [43].

#### Survival analysis

Follow-up began on the date of the last questionnaire used in the BMI modeling if the woman was postmenopausal or at the date of menopause if it occurred later. Women were followed until cancer diagnosis, death, emigration, or the end of the study (December 31, 2020), whichever occurred first. Cox proportional hazards models with attained age as the underlying time metric were used to estimate hazard ratios (HRs) and 95% confidence intervals (CIs) for the estimated BMI variables (overweight/obesity duration, intensity and age at onset, and trajectories of BMI) in relation to the overall, ER/PR-, and ER/PR/HER2-defined subtypes of postmenopausal breast cancer. For the intrinsic-like subtypes, we additionally modeled the BMI at wave 1. Separate regression models were constructed for each subtype outcome, censoring women who developed a subtype other than the one defined as failure at the time of diagnosis [44]. We fitted two models per outcome: age-adjusted and multivariable-adjusted. Participants with missing information on the included covariates were excluded from the multivariable-adjusted analysis. The included women were of different ages at their first enrollment into the NOWAC study. Thus, their total follow-up time and their possible time spent with overweight or obesity varied according to age at enrollment. To account for these differences, the regression models for overweight/obesity duration, intensity, and age at onset included age at enrollment in 10-year age groups as stratum variables. This allowed the baseline hazard to vary across age strata while keeping the coefficients equal across groups. The HRs for breast cancer subtypes were tested for heterogeneity by competing risk analyses using the data duplication method and likelihood ratio tests as described by Lunn and McNeil [45, 46]. All *p*-values were two-sided. The proportional hazards assumption was evaluated by testing Schoenfeld residuals and by graphically inspecting a log–log survival plot. All analyses were performed using the statistical package STATA version 17.0 (StataCorp, College Station, TX, USA).

## Results

During 2,221,544 person-years of follow-up, 7223 cases of incident invasive postmenopausal breast cancer occurred. Average follow-up time was 14.9 years (standard deviance [SD] 0.02). Changes in BMI were modeled over a range of 3–58 years, with a mean modeling duration of 36 years (SD 8.74).

**Fig. 1:**
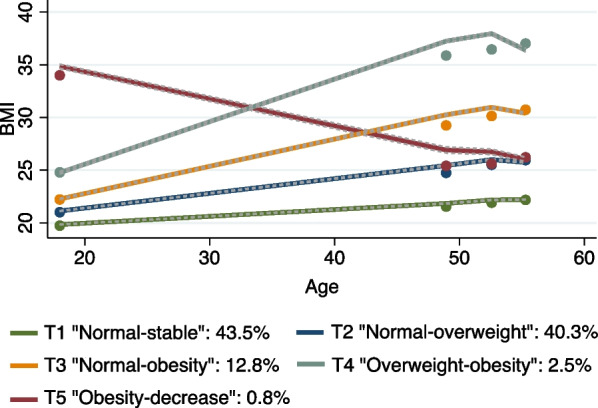
BMI trajectories (T1 “Normal-stable”; T2 “Normal-overweight”; T3 “Normal-obesity”; T4 “Overweight-obesity”; T5 “Obesity-decrease”) with 95% CI

Five distinct BMI trajectories were identified (Fig. 1): 43.5% of women had a consistent normal BMI (T1 “Normal-stable”); 40.3% started with normal weight and developed overweight in late adult life (T2 “Normal-overweight”); 12.8% evolved from normal to overweight in early adult life and had obesity in late adulthood (T3 “Normal-obesity”); 2.5% progressed from overweight to obesity (T4 “Overweight-obesity”); and 0.8% had a descending curve from obesity to overweight (T5 “Obesity-decrease”).

The individual trajectories for each group are depicted in Additional file 1: Fig. 2.

### Study sample characteristics

Compared with the “Normal-stable” (T1) group, the groups with increasing BMI (T2–T4) were less likely to have used exogenous hormones and to be physically active at wave 1 (Table 1).

The age at onset of overweight and obesity decreased, and the overweight duration increased from group T2 to T5. Apart from these differences, the characteristics of T1–T4 were relatively similar. Women in the “Obesity-decrease” (T5) group were more likely to be postmenopausal, never users of oral contraceptives, current users of MHT, current smokers, and less educated compared with the other trajectory groups. They also had higher physical activity levels than the trajectory groups who experienced weight gain (T2–T4).

### Characteristics of cancer cases

Of the 7223 incident invasive breast cancer cases, 5674 ER + (86.8%), 866 ER− (13.2%), 4379 PR + (67.4%), 2114 PR− (32.6%), 719 HER2 + (12.5%), and 5032 HER2− (87.5%) cases were identified (Table 2).

**Table 2:** Characteristics of postmenopausal breast cancer cases according to trajectory group

Postmenopausal breast cancer cases	T1 «Normal- stable»^a^	T2 «Normal-overweight»^a^	T3 «Normal- obesity»^a^	T4 «Overweight- obesity»^a^	T5 «Obesity- decrease»^a^
(*n* = 7223)	Mean ± SD or *n* (%)	Mean ± SD or *n* (%)	Mean ± SD or *n* (%)	Mean ± SD or *n* (%)	Mean ± SD or *n* (%)
**Cancer characteristics**					
Number of cases	3166 (43.8)	2987 (41.4)	885 (12.3)	145 (2.0)	40 (0.6)
Age at diagnosis	63.0 ± 0.13	63.3 ± 0.13	63.7 ± 0.23	62.2 ± 0.52	67.0 ± 1.31
*ER/PR status*					
ER+PR+	1805 (57.0)	1820 (60.9)	591 (66.8)	94 (64.8)	19 (47.5)
ER−PR−	376 (11.9)	327 (11.0)	91 (10.3)	16 (11.0)	4 (10.0)
ER+PR−	614 (19.4)	517 (17.3)	145 (16.4)	16 (11.0)	7 (17.5)
ER−PR+	26 (0.8)	18 (0.6)	1 (0.1)	2 (1.4)	1 (2.5)
Missing	345 (10.9)	305 (10.2)	57 (6.4)	17 (11.7)	9 (22.5)
*ER/PR/HER2 status*^*b*^					
Luminal A-like	1479 (46.7)	1496 (50.1)	481 (54.4)	78 (53.8)	15 (37.5)
Luminal B-like	634 (20.0)	557 (18.7)	167 (18.9)	21 (14.5)	8 (20.0)
HER2+	112 (3.5)	104 (3.5)	25 (2.8)	6 (4.1)	1 (2.5)
TNBC	212 (6.7)	184 (6.2)	58 (6.6)	9 (6.2)	3 (7.5)
Missing	729 (23.0)	646 (21.6)	154 (17.4)	31 (21.4)	13 (32.5)

*BMI* body mass index, *ER* estrogen receptor, *HER2* human epidermal growth factor receptor 2, *MHT* menopausal hormone therapy, *OC* oral contraceptives, *PR* progesterone receptor, *SD* standard deviation

^a^Normal weight: 18.5–24.9 kg/m^2^; overweight: 25–29.9 kg/m^2^; obesity: ≥ 30 kg/m^2^

^b^Luminal A-like: ER+PR + HER2−; luminal B-like: ER+PR− HER2− or ER+PR− HER2+or ER+PR+HER2+; HER2-enriched: ER- PR- HER2+; TNBC: ER- PR- HER2-

The number of missing cases were 683 (9.5%) for ER status, 730 (10.1%) for PR status, and 1472 (20.4%) for HER2 status. Missing values comprised a higher proportion in the T5 trajectory group but otherwise did not differ considerably across trajectory groups.

### Postmenopausal breast cancer overall

Increasing age at overweight was associated with increased risk of postmenopausal breast cancer (*p* trend < 0.01) and belonging to the “Obesity-decrease” trajectory group decreased risk of breast cancer (HR 0.71; 95% CI 0.52–0.96; Table 3).

**Table 3:** Age-adjusted and multivariable-adjusted hazard ratios for the association between body fatness and postmenopausal breast cancer overall

	Study sample (*n* = 148,866)	Cases	Age-adjusted HR (95% CI)	Complete-case study sample (*n* = 142,771)	Cases	MV-adjusted HR (95% CI)^a^
		(*n* = 7223)			(*n* = 6933)	
**Age at onset (yrs)**^*b*^						
*BMI ≥ 25*						
Never OW	83,606	3939	Ref	79,966	3766	Ref
< 40	26,375	1220	1.01 (0.95–1.08)	25,112	1173	1.00 (0.93–1.07)
40–49	25,208	1356	1.16 (1.09–1.23)	24,422	1311	1.14 (1.07–1.21)
≥ 50	13,677	708	1.18 (1.09–1.28)	13,271	683	1.16 (1.07–1.26)
*p*_trend_^c^			< 0.01			< 0.01
*BMI ≥ 30*						
Never OB	133,840	6504	Ref	128,327	6232	Ref
< 40	3242	120	0.80 (0.66–0.95)	3082	119	0.81 (0.68–0.97)
40–49	5858	286	1.02 (0.91–1.15)	5640	282	1.02 (0.91–1.15)
≥ 50	5926	313	1.17 (1.05–1.32)	5722	300	1.14 (1.02–1.29)
*p*_trend_^c^			0.03			0.06
**Duration (per 10 yrs)**^*b*^						
BMI **≥** 25	65,260	3284	1.02 (1.00–1.04)	62,805	3167	1.02 (0.99–1.04)
BMI **≥** 30	15,026	719	0.99 (0.94–1.05)	14,444	701	0.99 (0.93–1.05)
**Intensity (per 100 units)**^*b*^						
OWY	65,260	3284	1.00 (0.95–1.06)	62,805	3167	1.00 (0.94–1.05)
OBY	15,026	719	0.92 (0.78–1.08)	14,444	701	0.92 (0.78–1.08)
**Trajectories**^*d, e*^						
Normal-stable	62,729	3031	Ref	65,507	3166	Ref
Normal-overweight	58,079	2864	1.03 (0.98–1.08)	60,440	2987	1.02 (0.96–1.07)
Normal-obesity	17,374	854	1.03 (0.95–1.11)	18,117	885	1.01 (0.94–1.09)
Overweight-obesity	3454	144	0.88 (0.74–1.04)	3609	145	0.88 (0.75–1.05)
Obesity-decrease	1135	40	0.70 (0.51–0.96)	1193	40	0.71 (0.52–0.96)
*p*_trend_^c^			0.45			0.28

*BMI* body mass index, *CI* confidence interval, *HR* hazard ratio, *MV* multivariable, *OBY* weighted cumulative obesity years, *OWY* weighted cumulative overweight years, *p* *p*-value

^a^Adjusted for age, age at menarche, parity, age at first birth, breast cancer in mother, smoking, MHT use

^b^Based on linear mixed-effects models

^c^*p* trend, continuous variable

^d^Based on group-based trajectory modeling

^e^Normal weight: 18.5–24.9 kg/m^2^; overweight: 25–29.9 kg/m^2^; obesity: ≥ 30 kg/m^2^

### Postmenopausal breast cancer by ER/PR/HER2 status

Compared with normal-weight women, women with overweight or obesity at wave 1 had an increased risk of luminal A-like cancer with HRs of 1.11 (95% CI 1.02–1.20) and 1.13 (95% CI 1.00–1.28), respectively (*p*_trend_ 0.01; Table 4).

**Table 4:** Multivariable-adjusted hazard ratios for the association between body fatness and ER/PR/HER2-defined subtypes of postmenopausal breast cancer

	Luminal A-like (*n* = 3400)		Luminal B-like (*n* = 1324)		HER2-enriched (*n* = 235)		TNBC (*n* = 450)		*p*_het_^b^
	Cases	MV-adjusted HR (95% CI)^a^	Cases	MV-adjusted HR (95% CI)^a^	Cases	MV-adjusted HR (95% CI)^a^	Cases	MV-adjusted HR (95% CI)^a^	
**BMI at wave 1**^*c, d*^									
Underweight	72	1.14 (0.90–1.44)	348	0.94 (0.63–1.40)	1	0.20 (0.03–1.40)	12	1.39 (0.78–2.48)	
Normal weight	2038	Ref	852	Ref	154	Ref	271	Ref	
Overweight	966	1.11 (1.02–1.20)	93	0.95 (0.84–1.08)	62	0.99 (0.73–1.33)	125	1.09 (0.88–1.35)	
Obesity	303	1.13 (1.00–1.28)	6	0.81 (0.66–1.01)	18	0.91 (0.55–1.50)	41	1.14 (0.81–1.59)	
*p*_trend_^e^		0.01		0.09		0.82		0.54	0.06
**Age at onset (yrs)**^*f*^									
*BMI ≥ 25*									
Never OW	1780	Ref	736	Ref	136	Ref	247	Ref	
< 40	609	1.10 (1.00–1.21)	212	0.90 (0.77–1.05)	39	0.90 (0.63–1.30)	83	1.03 (0.80–1.33)	
40–49	698	1.27 (1.16–1.39)	230	1.00 (0.86–1.16)	46	1.11 (0.79–1.56)	74	0.95 (0.73–1.24)	
≥ 50	313	1.04 (0.92–1.18)	146	1.18 (0.99–1.42)	14	0.68 (0.39–1.19)	46	1.12 (0.81–1.55)	
*p*_trend_^e^		< 0.01		0.14		0.41		0.66	0.03
*BMI ≥ 30*									
Never OB	3024	Ref	1199	Ref	216	Ref	403	Ref	
< 40	63	0.90 (0.70–1.16)	18	0.63 (0.39–1.00)	5	0.96 (0.39–2.34)	7	0.70 (0.33–1.47)	
40–49	145	1.10 (0.93–1.30)	51	0.95 (0.72–1.26)	11	1.11 (0.61–2.06)	22	1.20 (0.78–1.86)	
≥ 50	168	1.23 (1.05–1.44)	56	1.03 (0.79–1.35)	3	0.34 (0.11–1.05)	18	0.98 (0.61–1.58)	
*p*_trend_^e^		< 0.01		0.87		0.12		0.87	0.29
**Duration (per 10 yrs)**^*f*^									
BMI **≥** 25	1620	1.04 (1.00–1.07)	588	0.93 (0.88–0.99)	99	0.98 (0.86–1.12)	203	0.98 (0.89–1.08)	0.03
BMI **≥** 30	376	1.01 (0.93–1.10)	125	0.88 (0.76–1.03)	19	0.95 (0.67–1.34)	47	0.96 (0.76–1.22)	0.56
**Intensity (per 100 units)**^*f*^									
OWY	1620	1.03 (0.96–1.11)	588	0.85 (0.74–0.97)	99	0.96 (0.70–1.30)	203	0.97 (0.78–1.20)	0.16
OBY	376	0.94 (0.75–1.18)	125	0.61 (0.38–0.99)	19	0.82 (0.31–2.15)	47	1.08 (0.63–1.86)	0.35
**Trajectories**^*g*^									
Normal-stable	1413	Ref	601	Ref	107	Ref	205	Ref	
Normal-overweight	1432	1.09 (1.01–1.18)	535	0.95 (0.84–1.06)	96	0.96 (0.73–1.27)	176	0.91 (0.74–1.11)	0.16
Normal-obesity	463	1.20 (1.08–1.33)	159	0.94 (0.78–1.12)	25	0.82 (0.53–1.28)	57	0.96 (0.72–1.30)	0.09
Overweight-obesity	77	1.05 (0.84–1.33)	21	0.64 (0.41–1.00)	6	1.00 (0.44–2.30)	9	0.78 (0.40–1.53)	0.3
Obesity-decrease	15	0.54 (0.33–0.90)	8	0.67 (0.33–1.35)	1	0.54 (0.08–3.88)	3	0.76 (0.24–2.37)	0.94
*p*_trend_^e^		0.07		0.05		0.42		0.37	

*BMI* body mass index, *CI* confidence interval, *HR* hazard ratio, *HER2* human epidermal growth factor receptor 2, *MV* multivariable, *OBY* weighted cumulative obesity years, *OWY* weighted cumulative overweight years, *p* *p*-value, *TNBC* triple-negative breast cancer

^a^Adjusted for age, age at menarche, parity, age at first birth, breast cancer in mother, smoking, MHT use

^b^*p* heterogeneity between ER/PR/HER2-defined subtypes; likelihood ratio test by competing risks analysis

^c^Number of missing values: 21 luminal A-like (0.6%); 6 luminal B-like (0.5%); 0 HER2-enriched; 1 TNBC (0.2%)

^d^Underweight: < 18.5 kg/m^2^; normal weight: 18.5–24.9 kg/m^2^; overweight: 25–29.9 kg/m^2^; obesity: ≥ 30 kg/m^2^

^e^*p* trend, continuous variable

^f^Based on linear mixed effects models. Never overweight/obesity as reference group

^g^Based on group-based trajectory modeling

Increased age at overweight and obesity onset was associated with an increased risk of luminal A-like cancer (*p* linear trend < 0.01). Increasing overweight duration increased the risk of luminal A-like cancer (HR per 10-year increment 1.04; 95% CI 1.00–1.07) and decreased the risk of luminal B-like cancer (HR per 10-year increment 0.93; 95% CI 0.88–0.99). Significant heterogeneity was observed across the subtypes with regard to overweight duration and age at overweight (*p*_heterogeneity_ 0.03). The HRs were similar to those of overweight/obesity duration when modeling weighted cumulative years of overweight/obesity for luminal A-like cancer. However, for luminal B-like cancer, HRs of 0.85 (95% CI 0.91–0.99) and 0.61 (95% CI 0.38–0.99) were observed for weighted cumulative years of overweight and obesity, respectively. Compared with the “Normal-stable” trajectory, women with constantly increasing BMI during adult life experienced an increased risk for luminal A-like cancer (HR 1.09; 95% CI 1.01–1.17 for “Normal-overweight”; HR 1.20; 95% CI 1.07–1.33 for “Normal-obesity”), whereas those with decreasing weight experienced a nearly 50% reduced risk (HR 0.54; 95% CI 0.33–0.90 for “Obesity-decrease”). With borderline-significance, the “Overweight-obesity” trajectory was associated with decreased risk of luminal B-like breast cancer (HR 0.64; 95% CI 0.41–1.00). No significant associations were observed for HER2-enriched or TNBC subtypes. Results of the age-adjusted analyses are provided in Additional file 1: Table 2.

### Postmenopausal breast cancer by ER/PR status and MHT use

The ER/PR-positive breast cancer results were similar to those for the luminal A-like subtype. Body fatness was positively associated with ER/PR-positive breast cancer, whereas we observed no significant association with ER/PR-negative cancer. Specifically, increased age at overweight and obesity onset was associated with ER/PR-positive breast cancer (*p*_trend_ < 0.01; Additional file 1: Table 3). We also observed an increased risk of ER/PR-positive breast cancer by overweight duration (HR per 10-year increment 1.05; 95% CI 1.02–1.08). The weighted cumulative years of overweight and obesity over time did not significantly change the risk of ER/PR-defined breast cancer. Compared with women belonging to the “Normal-stable” trajectory, women in the “Normal-overweight” and “Normal-obesity” trajectories had increased risk of ER/PR-positive breast cancer, with respective HRs of 1.09 (95% CI 1.01–1.16) and 1.19 (95% CI 1.08–1.31). Significant heterogeneity between ER/PR-positive and -negative breast cancer was observed for the HRs of the “Normal-obesity” trajectory (*p*_heterogeneity_ 0.03). Women in the “Obesity-decrease” trajectory had a 43% reduced risk of ER/PR-positive breast cancer (HR 0.57; 95% CI 0.36–0.90). Age-adjusted analyses yielded similar results.

Stratified analyses of MHT use suggested some extent of effect modification by MHT on the association between body fatness and ER/PR-positive breast cancer (Table 5). Significant associations were seen for ER/PR-positive breast cancer in never MHT users, and not in ever users. Specifically, in women who never used MHT, increased age at overweight and obesity onset increased the risk of ER/PR-positive breast cancer (*p*_trend_ < 0.01). Overweight duration per 10 years and weighted cumulative overweight years per 100 unit increase were associated with ER/PR-positive breast cancer (HR 1.09; 95% CI 1.04–1.13 and HR 1.10; 95% CI 1.01–1.19, respectively). Ascending trajectories from normal BMI were associated with ER/PR-positive breast cancer, where the “Normal-obesity” trajectory increased risk by 34% (95% CI 1.18–1.52). Women belonging to the descending trajectory appeared to be at 59% decreased risk (HR 0.41, 95% CI 0.20–0.87). Age at overweight onset (*p*_heterogeneity_ 0.04), overweight duration (*p*_heterogeneity_ 0.04) and the “Normal-overweight” (*p*_heterogeneity_ 0.01) and “Normal-obesity” (*p*_heterogeneity_ 0.01) trajectory were differentially associated with ER/PR-positive and ER/PR-negative breast cancer among never MHT users.

**Table 5:** Multivariable-adjusted hazard ratios for the association between body fatness and ER/PR-defined subtypes of postmenopausal breast cancer by MHT use

	Ever MHT use^a^					Never MHT use^a^				
	ER + /PR + (*n* = 1956)		ER-/PR- (*n* = 342)		*p*_het_^c^	ER + /PR + (*n* = 2230)		ER-/PR- (*n* = 447)		*p*_het_^c^
	Cases	MV-adjusted HR (95% CI)^b^	Cases	MV-adjusted HR (95% CI)^b^		Cases	MV-adjusted HR (95% CI)^b^	Cases	MV-adjusted HR (95% CI)^b^	
**Age at onset (yrs)**^*d*^										
*BMI ≥ 25*										
Never OW	1046	Ref	182	Ref		1151	Ref	261	Ref	
< 40	283	0.95 (0.83–1.08)	47	0.89 (0.64–1.23)		469	1.22 (1.09–1.36)	89	1.03 (0.80–1.31)	
40–49	392	1.13 (1.00–1.27)	68	1.11 (0.84–1.47)		455	1.37 (1.22–1.52)	67	0.90 (0.69–1.19)	
≥ 50	235	1.04 (0.90–1.20)	45	1.18 (0.85–1.64)		155	1.10 (0.93–1.31)	30	1.00 (0.68–1.48)	
*p*_trend_^e^		0.21		0.26	0.85		< 0.01		0.74	0.04
*BMI ≥ 30*										
Never OB	1775	Ref	312	Ref		1946	Ref	403	Ref	
< 40	22	0.73 (0.48–1.11)	3	0.56 (0.18–1.74)		56	1.00 (0.77–1.31)	9	0.77 (0.40–1.50)	
40–49	66	0.92 (0.72–1.17)	15	1.13 (0.67–1.91)		115	1.26 (1.04–1.52)	21	1.12 (0.72–1.75)	
≥ 50	93	1.02 (0.83–1.26)	12	0.76 (0.43–1.36)		113	1.56 (1.29–1.89)	14	0.99 (0.58–1.70)	
*p*_trend_^e^		0.81		0.45	0.64		< 0.01		0.92	0.35
**Duration (per 10 yrs)**^*d*^										
BMI **≥** 25	910	1.00 (0.96–1.04)	160	0.98 (0.88–1.09)	0.92	1,079	1.09 (1.05–1.13)	186	0.98 (0.89–1.08)	0.04
BMI **≥** 30	181	0.96 (0.85–1.08)	30	0.91 (0.67–1.23)	0.83	284	1.06 (0.97–1.17)	44	0.97 (0.77–1.24)	0.47
**Intensity (per 100 units)**^*d*^										
OWY	910	0.95 (0.85–1.06)	160	0.93 (0.71–1.22)	0.99	1,079	1.10 (1.01–1.20)	186	0.95 (0.76–1.19)	0.21
OBY	181	0.88 (0.62–1.24)	30	1.05 (0.50–2.17)	0.63	284	0.98 (0.76–1.26)	44	0.89 (0.48–1.68)	0.77
**Trajectories**^*f, g*^										
Normal-stable	874	Ref	157	Ref		864	Ref	207	Ref	
Normal-overweight	822	1.04 (0.94–1.14)	146	1.02 (0.81–1.28)	0.92	943	1.14 (1.04–1.26)	169	0.86 (0.70–1.05)	0.01
Normal-obesity	222	1.02 (0.88–1.18)	33	0.82 (0.56–1.20)	0.33	349	1.34 (1.18–1.52)	57	0.91 (0.67–1.22)	0.01
Overweight-obesity	26	0.77 (0.52–1.14)	5	0.79 (0.32–1.93)	0.93	67	1.20 (0.93–1.54)	11	0.81 (0.44–1.49)	0.23
Obesity-decrease	12	0.78 (0.44–1.37)	1	0.39 (0.05–2.76)	0.44	7	0.41 (0.20–0.87)	3	0.79 (0.25–2.47)	0.39
*p*_trend_^e^		0.64		0.26			<0.01		0.23	

*CI* confidence interval, *HR* hazard ratio, *ER* estrogen receptor, *MHT* menopausal hormone therapy, *MV* multivariable, *OBY* weighted cumulative obesity years, *OWY* weighted cumulative overweight years, *p* *p*-value

^a^Based on last reported MHT status prior to censoring

^b^Adjusted for age, age at menarche, parity, age at first birth, breast cancer in mother, smoking, physical activity

^c^*p* heterogeneity between ER + /PR + and ER−/PR− subtype; likelihood ratio test by competing risks analysis

^d^Based on linear mixed effects models. Never overweight/obesity as reference group

^e^*p* trend, continuous variable

^f^Based on group-based trajectory modeling

^g^Normal weight: 18.5–24.9 kg/m^2^; overweight: 25–29–9 kg/m^2^; obesity: ≥ 30 kg/m^2^

## Discussion

In this exploratory study, we assessed the relationship between BMI trajectories in adult life, duration, intensity, and onset of body fatness and subtypes of postmenopausal breast cancer in a large national cohort of Norwegian women. We observed that obese women who decreased their weight had a reduced risk of hormone receptor-positive or luminal A-like breast cancer compared with women who remained at normal weight throughout their adult life. Adult overweight duration, increased age at onset of overweight or obesity, and ascending BMI trajectories throughout adulthood were associated with an increased risk of hormone receptor-positive and luminal A-like breast cancer. Similar findings were observed in postmenopausal breast cancer overall, likely because the luminal A-like subtype constitutes the largest proportion of breast cancer cases in postmenopausal women. Significant associations between body fatness and hormone receptor-positive breast cancer were predominantly evident in never users of MHT. The findings regarding BMI trajectories are novel and highlight the potential health benefits of weight reduction among adult women with obesity and the health risks associated with consistent weight gain.

Our study aligns with the existing literature revealing that body fatness in adulthood is associated with hormone receptor-positive or luminal A-like tumors [12, 14–22]. A recent prospective study revealed significant associations between postmenopausal obesity and luminal A-like breast cancer, whereas no significant association was observed with either luminal B, HER2-enriched, or TNBC [19]. Furthermore, a German arm of the European Prospective Investigation into Cancer study demonstrated that a higher BMI was associated with luminal A-like breast cancer in postmenopausal women but not with aggressive tumor subtypes [21]. However, controversies exist; while some prospective studies have observed no associations between body fatness and hormone-receptor negative cancer in postmenopausal women [14, 15, 17, 19–21], other studies have reported positive associations [47–50]. For TNBC, case–control studies have observed both positive [48, 49] and inverse [51] associations with body fatness in postmenopausal women. Thus, results are inconsistent as to whether there is an association of BMI and estrogen receptor-negative breast cancer. We did not find previous reports suggesting inverse associations between body fatness and luminal B-like breast cancer as we did for overweight duration, intensity, and weight gain from overweight. To our knowledge, most studies have reported non-significant results [8, 19, 52, 53], and one study reported an increased risk of the luminal B-like subtype among women with obesity compared with normal-weight women [14]. Variations in the study design, age, measure of exposure, sample size, and subtype definition may explain these discrepancies. Of note, the findings for luminal B-like breast cancer need to be interpreted with caution due to low statistical power and thus the possibility that they were made by chance.

While many studies have addressed weight change in relation to the risk of subtypes of breast cancer [8, 16, 17, 20, 54–57], to our knowledge, this is the first study to assess associations between BMI trajectories and breast cancer subtypes. Previous studies on life-course fluctuations of body fatness in relation to breast cancer risk used trajectories of perceived body silhouettes [28–31]. This measure of body fatness may be more prone to misclassification, especially among heavier, shorter, younger, and less educated women [58]. Furthermore, these previous studies started the trajectory modeling in childhood and focused on the mechanisms of pre-pubertal body fatness and breast cancer. As we did not obtain BMI measurements in childhood, we could not extend our modeling to a complete life-course perspective of BMI development.

The mechanisms underlying the association between body fatness and hormone receptor-positive breast cancer in postmenopausal women involve hormonal pathways. Increased circulating levels of bioavailable estrogen are observed with increasing body fatness in postmenopausal women because adipose tissue remains the major site of aromatase activity after menopause, together with reduced production of sex hormone-binding globulin and alterations in androgen metabolism [59–61]. Indeed, women with normal BMI and high body fat percentages have a higher risk of postmenopausal breast cancer [62]. Other studies have revealed that increased estrogen levels largely explain the association between BMI and postmenopausal breast cancer [13]. While the association with luminal A-like breast cancer is evident, similar associations were not observed for luminal B-like breast cancer in the present work. Luminal B-like cancers have a lower expression of the PR protein than luminal A-like cancers [63], which may reflect the importance of the interaction between ER and PR [64]. Hormone receptor-negative subtypes are less prone to estrogen influence, which may explain why we did not observe a significant association with these subtypes. Other potential contributing mechanisms include altered insulin and insulin-like growth factor-I levels and chronic low-grade inflammation [65]. It is not unlikely that body fatness duration and timing influence these key mechanisms.

Our study is consistent with previous reports illustrating that the risk of postmenopausal breast cancer related to body fatness is modified by MHT use [18, 20, 26, 36, 47, 66–69]. We observed that associations between body fatness and hormone receptor-positive breast cancer were largely eliminated in ever-users of MHT. Moreover, overweight intensity significantly increased the risk of hormone receptor-positive subtypes in never-users. A proposed mechanism underlying this phenomenon is the obscuring effect of high exogenous estrogen intake from MHT, leaving relatively negligible endogenous estrogen levels at high BMI.

This study had several limitations. Despite the large sample size, we were restricted by lack of power in the subgroup analyses of the less common subtypes. Under the assumption that receptor status data were missing at random, we chose not to perform multiple imputation in order to maintain transparency and simplicity [70]. Moreover, self-reported weight tends to be underestimated with increasing age and BMI [71], as revealed by the validity assessment in the NOWAC study [72]. However, a substantial agreement was observed between the self-reported values and those measured by medical staff (weighted kappa = 0.73). The 8156 participants who were excluded due to having less than two BMI measurements and the 6697 participants excluded due to missing physical activity or smoking status at all time points differed slightly from the total study sample with regard to age, BMI and education (Additional file 1: Table 1). Hence, the exclusions may have resulted in a slightly slimmer study sample compared to all NOWAC participants since women with high BMI seem to be less likely to repeatedly report their BMI. Reporting bias or misclassification of included covariates may have resulted in residual confounding. Of note, breast density is a potential effect modifier on the association between BMI and breast cancer [50, 73, 74]. Unfortunately, we did not have information about the study participants´ breast density and hence, could not take that into account. Finally, due to the exploratory nature of this study, we did not adjust for multiple testing and results must be interpreted as such [75].

## Conclusion

Our exploratory study suggests that decreasing body fatness from obesity in adulthood is inversely associated with overall, hormone receptor-positive and luminal A-like breast cancer in postmenopausal women. Positive associations were observed with increasing body fatness from normal BMI during adulthood. Furthermore, we demonstrated a dose–response relationship between overweight duration and these subtypes, with significant heterogeneity between the intrinsic-like subtypes. As breast cancer is the most frequently diagnosed malignancy in women, and the prevalence of body fatness is increasing, preventive measures, such as weight loss, could contribute to halting an undue increase in breast cancer incidence following the obesity epidemic.

### Supplementary Information

**Additional file 1. Table 1.** Comparison of selected characteristics at wave 1 of excluded participants vs. study sample. **Table 2.** Age-adjusted hazard ratios for the association between body fatness and ER/PR/HER2-defined subtypes of postmenopausal breast cancer. **Table 3.** Age-adjusted and multivariable-adjusted hazard ratios for the association between body fatness and ER/PR-defined subtypes of postmenopausal breast cancer. **Figure 1.** Directed acyclic graph on the assumed relations between BMI development in adulthood, postmenopausal breast cancer and covariates. **Figure 2.** Twoway scatterplots of individual BMI trajectories by trajectory group.

## Data Availability

The datasets used and/or analyzed in this study are available from the corresponding author upon reasonable request and if legal permissions are in place.

## Abbreviations

BMI: Body mass index
CI: Confidence interval
ER: Estrogen receptor
HR: Hazard ratio
MHT: Menopausal hormone therapy
NOWAC: The Norwegian Women and Cancer Study
PR: Progesterone receptor
