# Supplementary material for: Trajectories of body mass index in adulthood and risk of subtypes of postmenopausal breast cancer
Source: Breast Cancer Res. 2023 Oct 28;25:130. doi: 10.1186/s13058-023-01729-x (PMC10612168; doi:10.1186/s13058-023-01729-x)
Supplement: Supplementary file 1 — Additional file 1. Table 1. Comparison of selected characteristics at wave 1 of excluded participants vs. study sample. Table 2. Age-adjusted hazard ratios for the association between body fatness and ER/PR/HER2-defined subtypes of postmenopausal breast cancer. Table 3. Age-adjusted and multivariable-adjusted hazard ratios for the association between body fatness and ER/PR-defined subtypes of postmenopausal breast cancer. Figure 1. Directed acyclic graph on the assumed relations between BMI development in adulthood, postmenopausal breast cancer and covariates. Figure 2. Twoway scatterplots of individual BMI trajectories by trajectory group. [file 13058_2023_1729_MOESM1_ESM.docx]

# Supplementary file

**Supplementary Table 1**. Comparison of selected characteristics at wave 1 of excluded participants vs. study sample

|  | Study sample (n = 148,866) | Excluded due to less than 2 BMI measurements (n = 8,156) | Excluded due to missing physical activity on all time points (n = 6,206) | Excluded due to missing smoking status on all time points (n = 820) |
| --- | --- | --- | --- | --- |
| Age, mean ± SD | 49.1 (0.02) | 53.7 (0.09) | 53.7 (0.11) | 51.2 (0.31) |
| BMI, mean ± SD  Age 18  Wave 1  Wave 2  Wave 3 | 20.8 (0.01)  24.2 (0.01)  24.9 (0.01)  25.3 (0.02) | 21.1 (0.11)  25.1 (0.06)  26.4 (0.19)  26.7 (0.73) | 21.3 (0.05)  25.1 (0.05)  26.0 (0.10)  26.8 (0.42) | 20.9 (0.11)  24.8 (0.14)  24.7 (0.50)  23.8 (0.58) |
| Parity, mean ± SD | 2.2 (0.00) | 2.2 (0.02) | 2.4 (0.02) | 2.3 (0.04) |
| Smoking status, %  Current  Former  Never | 34.7  34.6  30.7 | 37.4  32.8  29.8 | 38.8  28.0  33.2 | N/A  N/A  N/A |
| Physical activity, %  High  Moderate  Low | 18.2  57.4  24.4 | 20.9  54.9  24.2 | N/A  N/A  N/A | 19.1  55.5  25.5 |
| Education, %  ≤ 9  10-12  13-16  ≥ 17 | 21.8  34.4  28.5  15.4 | 37.0  33.3  19.4  10.2 | 53.1  29.2  12.7  5.1 | 35.7  35.3  19.6  9.5 |

Participants who were excluded due to less than 2 BMI measurements were older, had somewhat higher BMI and had lower education than the study sample. Participants who were excluded due to missing physical activity or smoking status on all time points were older, had somewhat higher BMI and had lower education than the study sample.

**Supplementary Table 2**. Age-adjusted hazard ratios for the association between body fatness and ER/PR/HER2-defined subtypes of postmenopausal breast cancer

|  | **Luminal A-like (n = 3,549)** | | **Luminal B-like (n = 1,387)** | | **HER2-enriched (n = 248)** | | **TNBC (n = 466)** | | ***p*_het_^a^** |
| --- | --- | --- | --- | --- | --- | --- | --- | --- | --- |
|  | **Cases** | **Age-adjusted HR (95% CI)^a^** | **Cases** | **Age-adjusted HR (95% CI)^a^** | **Cases** | **Age-adjusted HR (95% CI)^a^** | **Cases** | **Age-adjusted HR (95% CI)^a^** |  |
| **BMI at wave 1^b, c^**  Normal weight  Underweight  Overweight  Obesity | 2,140  74  1,001  311 | Ref.  1.10 (0.87-1.38)  1.13 (1.05-1.22)  1.16 (1.03-1.31) | 893  26  366  96 | Ref.  0.92 (0.62-1.36)  0.99 (0.88-1.12)  0.86 (0.70-1.07) | 163  2  64  18 | Ref.  0.37 (0.09-1.48)  1.00 (0.75-1.34)  0.93 (0.57-1.51) | 281  12  130  42 | Ref.  1.32 (0.74-2.36)  1.14 (0.92-1.40)  1.22 (0.88-1.69) | 0.13 |
| **Age at onset (yrs)^d^**  BMI ≥ 25  Never OW  < 40  40-49  ≥ 50  *p*_trend_^e^  BMI ≥ 30  Never OB  < 40  40-49  ≥ 50  *p*_trend_^e^ | 1,869  634  724  322  3,164  63  147  175 | Ref.  1.10 (1.01-1.20)  1.28 (1.17-1.39)  1.04 (0.93-1.18)  < 0.01  Ref.  0.87 (0.68-1.12)  1.09 (0.92-1.28)  1.25 (1.07-1.46)  < 0.01 | 772  224  236  155  1,256  19  51  61 | Ref.  0.94 (0.81-1.09)  1.01 (0.87-1.17)  1.23 (1.03-1.47)  0.06  Ref.  0.65 (0.42-1.03)  0.95 (0.72-1.26)  1.11 (0.86-1.44)  0.75 | 143  40  49  16  228  5  11  4 | Ref.  0.92 (0.65-1.30)  1.17 (0.84-1.62)  0.76 (0.45-1.29)  0.68  Ref.  0.95 (0.39-2.31)  1.12 (0.61-2.05)  0.44 (0.16-1.19)  0.20 | 255  85  78  48  419  7  22  18 | Ref.  1.08 (0.84-1.38)  1.02 (0.79-1.31)  1.17 (0.86-1.61)  0.37  Ref.  0.71 (0.34-1.50)  1.24 (0.80-1.90)  0.99 (0.62-1.59)  0.80 | 0.03  0.42 |
| **Duration (per 10 yrs)^d^**  BMI **≥** 25  BMI **≥** 30 | 1,680  385 | 1.04 (1.01-1.07)  1.01 (0.93-1.09) | 615  131 | 0.95 (0.90-1.00)  0.89 (0.77-1.03) | 105  20 | 1.00 (0.89-1.14)  0.95 (0.68-1.33) | 211  47 | 1.00 (0.92-1.09)  0.98 (0.78-1.23) | 0.03  0.55 |
| **Intensity (per 100 units)^d^**  OWY  OBY | 1,680  385 | 1.03 (0.96-1.11)  0.93 (0.75-1.16) | 615  131 | 0.87 (0.76-0.99)  0.62 (0.39-1.00) | 105  20 | 0.98 (0.73-1.31)  0.82 (0.32-2.15) | 211  47 | 0.99 (0.81-1.22)  1.13 (0.66-1.91) | 0.16  0.34 |
| **Trajectories^f^**  Normal-stable  Normal-overweight  Normal-obesity  Overweight-obesity  Obesity-decrease  *p*_trend_^e^ | 1,479  1,496  481  78  15 | Ref.  1.10 (1.02-1.18)  1.21 (1.09-1.34)  1.04 (0.83-1.30)  0.54 (0.32-0.89)  0.04 | 634  557  167  21  8 | Ref.  0.95 (0.85-1.07)  0.98 (0.82-1.16)  0.65 (0.42-1.00)  0.66 (0.33-1.32)  0.09 | 112  104  25  6  1 | Ref.  1.01 (0.78-1.32)  0.82 (0.53-1.27)  1.02 (0.45-2.32)  0.51 (0.07-3.67)  0.47 | 212  184  58  9  3 | Ref.  0.94 (0.77-1.15)  1.01 (0.75-1.35)  0.82 (0.42-1.60)  0.74 (0.24-2.33)  0.57 | 0.14  0.07  0.26  0.95 |
| Abbreviations: BMI: body mass index; CI: confidence interval; HR: hazard ratio; HER2: human epidermal growth factor receptor 2; TNBC: triple-negative breast cancer; OBY: weighted cumulative obesity years; OWY: weighted cumulative overweight years; p: p-value.  ^a^ *p* heterogeneity between ER/PR/HER2-defined subtypes; likelihood ratio test by competing risks analysis.  ^b^ Number of missing values: 23 luminal A-like (0.7%); 6 luminal B-like (0.4%); 1 HER2-enriched (0.4%); 1 TNBC (0.2%).  ^c^ Underweight: < 18.5 kg/m^2^; normal weight: 18.5–24.9 kg/m^2^; overweight: 25–29.9 kg/m^2^; obesity: ≥ 30 kg/m^2^.  ^d^ Based on linear mixed effects models. Never overweight/obesity as reference group.  ^e^ *p* trend, continuous variable.  ^f^ Based on group-based trajectory modeling. | | | | | | | | | |

**Supplementary Table 3**. Age-adjusted and multivariable-adjusted hazard ratios for the association between body fatness and ER/PR-defined subtypes of postmenopausal breast cancer

|  | **ER+/PR+ (n = 4,329)** | | **ER-/PR- (n = 814)** | | ***p*_het_^a^** | **ER+/PR+ (n = 4,150)** | | **ER-/PR- (n = 782)** | | ***p*_het_^a^** |
| --- | --- | --- | --- | --- | --- | --- | --- | --- | --- | --- |
|  | **Cases** | **Age-adjusted HR (95% CI)** | **Cases** | **Age-adjusted HR (95% CI)** |  | **Cases** | **MV-adjusted HR (95% CI)^b^** | **Cases** | **MV-adjusted HR (95% CI)^b^** |  |
| **Age at onset (yrs)^c^**  BMI ≥ 25  Never OW  < 40  40-49  ≥ 50  *p*_trend_^d^  BMI ≥ 30  Never OB  < 40  40-49  ≥ 50  *p*_trend_^d^ | 2,285  779  869  396  3,855  78  183  213 | Ref.  1.11 (1.02-1.20)  1.26 (1.17-1.37)  1.09 (0.98-1.22)  < 0.01  Ref.  0.88 (0.70-1.10)  1.11 (0.95-1.28)  1.30 (1.13-1.49)  < 0.01 | 456  139  140  79  740  12  36  26 | Ref.  0.99 (0.82-1.20)  1.04 (0.86-1.26)  1.18 (0.92-1.50)  0.22  Ref.  0.69 (0.39-1.22)  1.13 (0.81-1.59)  0.88 (0.59-1.30)  0.67 | 0.18  0.23 | 2,183  745  838  384  3,687  78  180  205 | Ref.  1.10 (1.01-1.20)  1.25 (1.16-1.36)  1.08 (0.97-1.21)  < 0.01  Ref.  0.91 (0.73-1.14)  1.12 (0.96-1.30)  1.28 (1.11-1.48)  < 0.01 | 439  136  133  74  709  12  36  25 | Ref.  0.98 (0.80-1.19)  0.99 (0.82-1.21)  1.11 (0.86-1.43)  0.54  Ref.  0.70 (0.39-1.23)  1.13 (0.81-1.58)  0.85 (0.57-1.27)  0.58 | 0.18  0.25 |
| **Duration (per 10 yrs)^c^**  BMI **≥** 25  BMI **≥** 30 | 2,044  474 | 1.05 (1.02-1.08)  1.02 (0.95-1.10) | 358  74 | 0.99 (0.93-1.07)  0.95 (0.79-1.14) | 0.15  0.45 | 1,967  463 | 1.05 (1.02-1.08)  1.03 (0.95-1.11) | 343  73 | 0.98 (0.91-1.05)  0.95 (0.79-1.14) | 0.14  0.53 |
| **Intensity (per 100 units)^c^**  OWY  OBY | 2,044  474 | 1.05 (0.98-1.12)  0.94 (0.77-1.15) | 358  74 | 0.96 (0.81-1.13)  0.95 (0.60-1.53) | 0.33  0.97 | 1,967  463 | 1.05 (0.98-1.12)  0.95 (0.78-1.17) | 343  73 | 0.95 (0.80-1.12)  0.95 (0.59-1.52) | 0.38  0.92 |
| **Trajectories^e, f^**  Normal-stable  Normal-overweight  Normal-obesity  Overweight-obesity  Obesity-decrease  *p*_trend_^d^ | 1,805  1,820  591  94  19 | Ref.  1.10 (1.03-1.17)  1.21 (1.10-1.33)  1.01 (0.82-1.25)  0.57 (0.36-0.89)  0.02 | 376  327  91  16  4 | Ref.  0.95 (0.82-1.10)  0.89 (0.71-1.11)  0.81 (0.49-1.33)  0.60 (0.22-1.60)  0.12 | 0.08  0.01  0.41  0.94 | 1,730  1,743  565  93  19 | Ref.  1.09 (1.01-1.16)  1.19 (1.08-1.31)  1.03 (0.83-1.27)  0.57 (0.36-0.90)  0.06 | 361  311  90  16  4 | Ref.  0.92 (0.79-1.07)  0.88 (0.70-1.11)  0.80 (0.48-1.32)  0.62 (0.23-1.66)  0.10 | 0.06  0.03  0.42  0.93 |
| Abbreviations: BMI: body mass index; CI: confidence interval; ER: estrogen receptor; HR: hazard ratio; MV: multivariable; OBY: weighted cumulative obesity years; OWY: weighted cumulative overweight years; p: p-value.  ^a^ *p* heterogeneity between ER+/PR+ and ER-/PR- subtype; likelihood ratio test by competing risks analysis.  ^b^ Adjusted for age, age at menarche, parity, age at first birth, breast cancer in mother, smoking, MHT use.  ^c^ Based on linear mixed effects models. Never overweight/obesity as reference group.  ^d^ *p* trend, continuous variable.  ^e^ Based on group-based trajectory modeling.  ^f^ Normal weight: 18.5–24.9 kg/m^2^; overweight: 25–29.9 kg/m^2^; obesity: ≥ 30 kg/m^2^. | | | | | | | | | | |

**Supplementary Figure 1**. Directed acyclic graph on the assumed relations between BMI development in adulthood, postmenopausal breast cancer and covariates

Created from <https://dagitty.net>. Based on the acyclic graph, we adjusted for a minimal sufficient adjustment set of variables to control for confounding, except for body size in childhood and alcohol consumption due to missing values. Adjustments were made for confounding factors depicted in white. The following assumptions were made when considering covariates for the multivariable model: 1) Age is related to body fatness as older women tend to be leaner than younger women, and various birth cohorts have different BMI development. Age is also a risk factor for breast cancer. We further assumed that age of women affected OC and MHT status; 2) Age at menarche could be related to weight in adulthood as well as breast cancer incidence. One study demonstrated that age at menarche is inversely associated with subsequent obesity^[[1]](#footnote-2)^; 3) Parity/age at first birth could be related to body fatness in addition to breast cancer incidence, as weight gained during pregnancy is not completely lost following delivery, leading to progressive weight gain over multiple pregnancies and, for some, development of obesity^[[2]](#footnote-3)^. Parity is also related to breastfeeding duration, alcohol consumption and smoking; 4) MHT use is a risk factor for breast cancer and could potentially cause weight change although no current evidence exists; 5) Breast cancer in mother is a proxy for genetic susceptibility for breast cancer (BRCA1/2 mutations) and could affect subsequent choices such as exogenous hormone use, age at first birth and parity; 6) Physical activity has a protective effect on breast cancer risk, and reductions in habitual levels of physical activity and increased sedentary behaviors is associated with increase risk of obesity^[[3]](#footnote-4)^. Vigorous physical activity could postpone pubertal onset; 7) Smoking cessation is associated with weight gain^[[4]](#footnote-5)^ as well as being a risk factor for breast cancer. Smoking behavior is related to physical activity and alcohol consumption.

**Supplementary Figure 2** – Twoway scatterplots of individual BMI trajectories by trajectory group


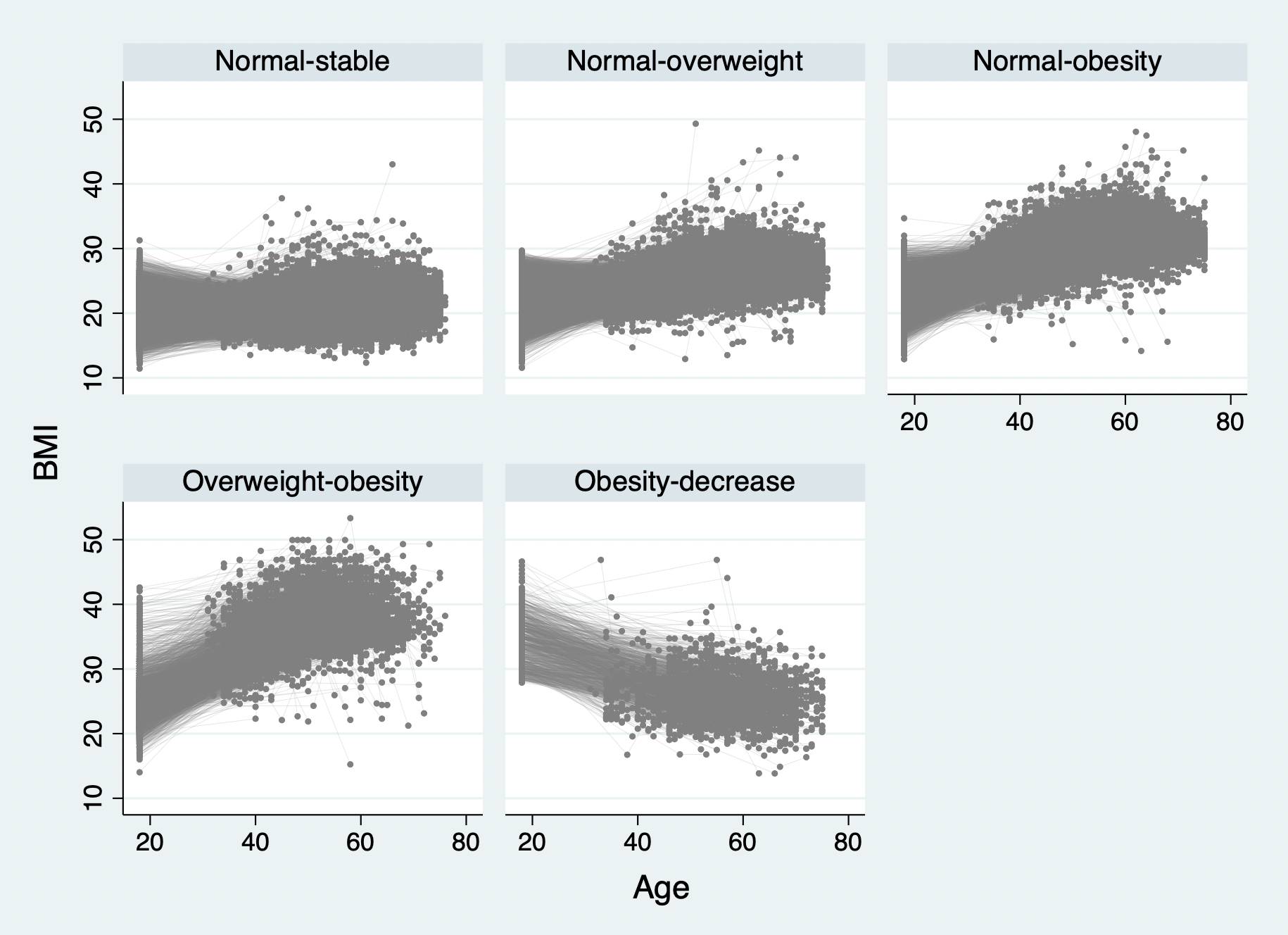


1. Yang L, Li L, Millwood IY, Lewington S, Guo Y, Sherliker P, et al. Adiposity in relation to age at menarche and other reproductive factors among 300 000 Chinese women: findings from China Kadoorie Biobank study. Int J Epidemiol. 2017;46(2):502-12 [↑](#footnote-ref-2)
2. Mannan M, Doi SA, Mamun AA. Association between weight gain during pregnancy and postpartum weight retention and obesity: a bias-adjusted meta-analysis. Nutr Rev. 2013;71(6):343-52. [↑](#footnote-ref-3)
3. Piercy KL, Troiano RP, Ballard RM, Carlson SA, Fulton JE, Galuska DA, et al. The Physical Activity Guidelines for Americans. JAMA. 2018;320(19):2020-8. [↑](#footnote-ref-4)
4. Bush T, Lovejoy JC, Deprey M, Carpenter KM. The effect of tobacco cessation on weight gain, obesity, and diabetes risk. Obesity (Silver Spring). 2016;24(9):1834-41. [↑](#footnote-ref-5)
